# Comparison of invasive ICP measurements to Distortion Product Otoacoustic Emissions (DPOAE) in adults during infusion testing for INPH

**DOI:** 10.1186/2045-8118-12-S1-O60

**Published:** 2015-09-18

**Authors:** MA Williams, SE Voss, NJ Horton, J Malm, A Eklund

**Affiliations:** 1 The Sandra and Malcolm Berman Brain & Spine Institute, USA; 2 Smith College, USA; 3 Amherst College, USA; 4 Umeå University, Sweden

## Introduction

Noninvasive ICP measurements are needed for astronauts at risk for the Visual Impairment/Intracranial Pressure (VIIP) syndrome. We evaluated distortion product otoacoustic emissions (DPOAE) for potential use to monitor ICP changes noninvasively.

## Methods

Eight subjects, mean age 68.5±7.4 years (range 58-79 years), undergoing lumbar CSF infusion testing (Likvor Celda, Umeå, Sweden) for hydrocephalus had DPOAE measurements made during ICP recording in the supine and upright positions, and during the infusion testing at 6 different ICP levels. DPOAE were measured with the HearID system (Mimosa Acoustics, Champaign, IL) at 13 log-spaced frequencies between 500 and 4000 Hz. DPOAE magnitudes within 6 dB of the noise floor, due in part to presbyacusis, were not analyzed, resulting in 5 of 13 frequencies with results that could be analyzed. Changes in DPOAE magnitudes and angles from the upright position were analyzed for these 5 frequencies at 7 ICP levels. For the DPOAE magnitudes and angles at each ICP and frequency, a 95% confidence interval was calculated using a bootstrap method. A statistically significant difference is present when the confidence interval does not contain zero.

## Results

In general, increasing ICP resulted in decreasing DPOAE magnitude and increasing DPOAE angle at all frequencies. DPOAE angles show statistically significant changes with ICP for all 5 frequencies, and the changes increase systematically with increasing ICP. Statistically significant changes in DPOAE magnitudes were also present, but for fewer frequencies and only at higher ICP levels.

## Conclusions

This is the first study to measure change in DPOAE magnitude and angle as a function of ICP. Systematic trends are present for both magnitude and angle; however DPOAE angle appears more robust, consistent with a previous study of DPOAE in patients undergoing LP and CSF removal. DPOAE may be more reliable in younger subjects with better hearing than the subjects evaluated in this study. For future use of DPOAE as a noninvasive ICP modality, if knowledge of the magnitude of ICP change (in mm Hg or kPa) in relation to change in DPOAE magnitude or angle is required, then an initial calibration of DPOAE with invasive ICP monitoring will be necessary.

## Acknowledgements

National Space Biomedical Research Institute Project #SMST02802

